# Supplementary material for: Mutation in the Common Docking Domain Affects MAP Kinase ERK2 Catalysis and Stability
Source: Cancers (Basel). 2023 May 26;15(11):2938. doi: 10.3390/cancers15112938 (PMC10252016; doi:10.3390/cancers15112938)
Supplement: Supplementary file 1 [file cancers-15-02938-s001.zip › cancers-2371633-supplementary.pdf]

Supplementary Material

Table S1. List and details of the analyzed ERK2 variants selected from COSMIC database

	Solvent Accessibility	Nucleotide substitution	Pathogenicity	Cancer Tissue
**D321A**	**Exposed**	**A > C**	**0.98**	**Liver**
**D321E**	**Exposed**	**C > G**	**0.85**	**Urinary tract**
**D321G**	**Exposed**	**A > G**	**0.97**	**Lung**
**D321V**	**Exposed**	**A > T**	**0.98**	**Skin**
**D321N**	**Exposed**	**G > A**	**0.98**	**Upper aerodigestive tract**
**E322K**	**Exposed**	**G > A**	**0.98**	**Liver**

The table reports the amino acid substitution (in red), the solvent accessibility, the information about nucleotide substitution, pathogenicity and cancer tissues that are reported in the COSMIC database [24]. The pathogenicity scores for individual mutations from FATHMM-MKL are in the form of a single p-value, ranging from 0 to 1. Scores above 0.5 are deleterious, but to highlight the most significant data in COSMIC, only scores ≥ 0.7 are classified as 'Pathogenic'. Mutations are classed as 'Neutral' if the score is ≤ 0.5.

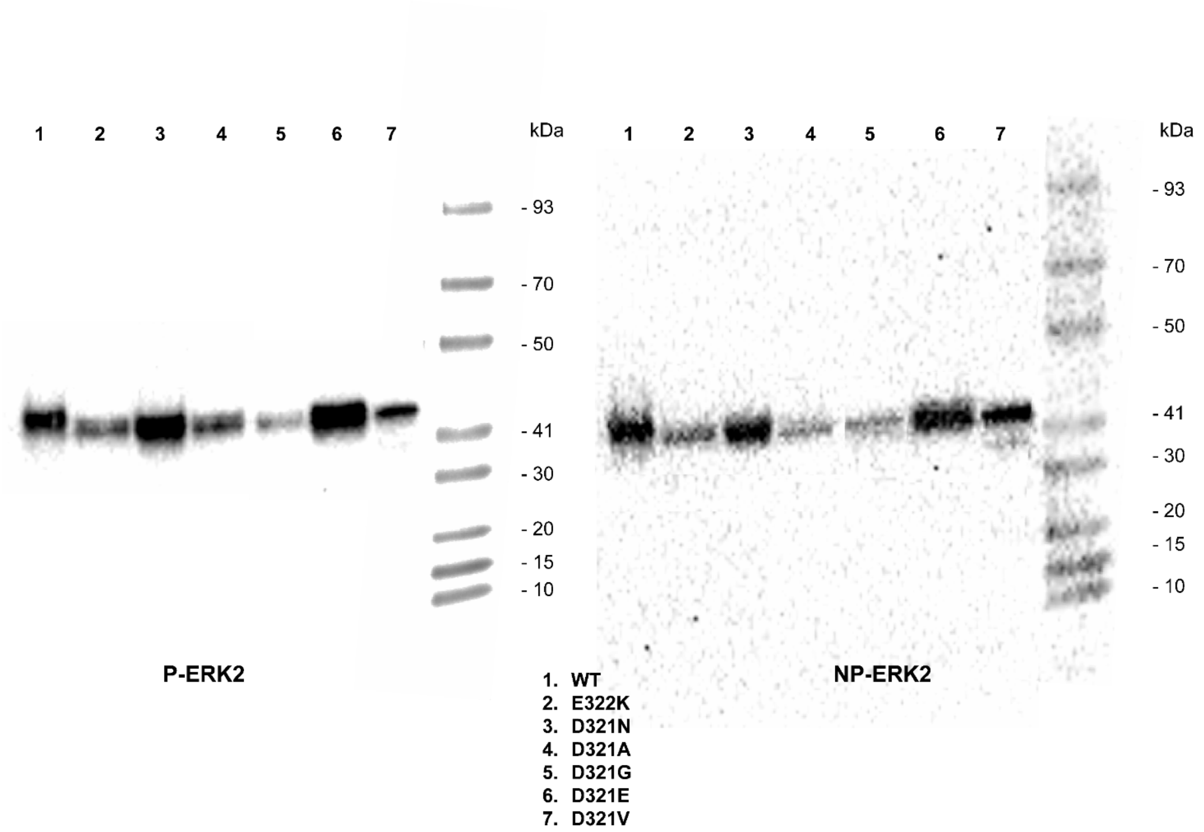

Figure S1. Western blot analysis of ERK2 wild-type and variants. The phosphorylation of ERK2 wild-type and variants were analyzed by Western blotting using the monoclonal antibody raised against the doubly phosphorylated ERK2 (anti-Phospho-ERK1/ERK2-Thr185, Tyr187) and the antibody anti-ERK1/ERK2, as described in Materials and Methods. NP-ERK2: non-phoshorylated ERK2; P-ERK2: phoshorylated ERK2.

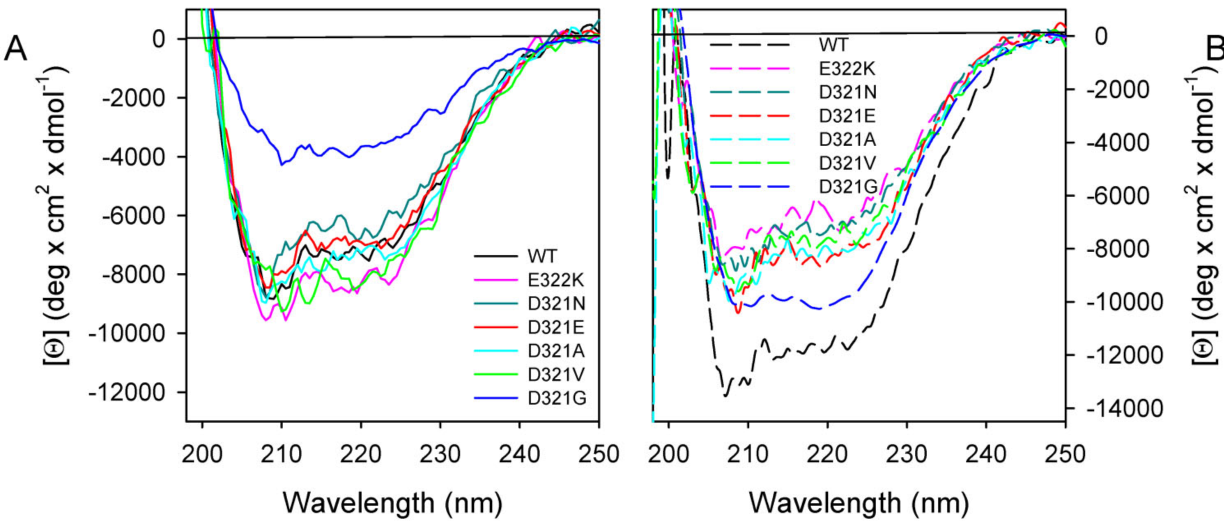

Figure S2. FAR-UV CD spectra of ERK2 wild-type (WT) and variants. (**A**) nonphosphorylated-ERK2 (NP-ERK2) WT and variants in the CD-site. (**B**) phosphorylated ERK2 (P-ERK2) WT and variants in the CD-site. All spectra were recorded at 20°C in a 0.1-cm quartz cuvette at 130-170 μg/mL in 20 mM Tris-HCl, pH 7.5 containing 0.2 M NaCl and 0.2 mM DTT.

Table S2. Molar ellipticity ratio of nonphosphorylated-ERK2 (NP-ERK2) and phosphorylated ERK2 (P-ERK2) wild type and variants.

	NP-ERK2	P-ERK2
**ERK2**	$[\Theta]_{222/208}$	$[\Theta]_{222/208}$
**wild-type**	0.80	0.90
**D321A**	0.80	0.82
**D321E**	0.81	0.83
**D321G**	1.11	0.98
**D321N**	0.76	0.83
**D321V**	1.01	0.77
**E322K**	0.81	0.90

The $[\Theta]_{222/208}$ ratio between 222 and at 208 nm is indicative of non-interacting helices (0.8-0.9) and coiled coil helices (>1.0).

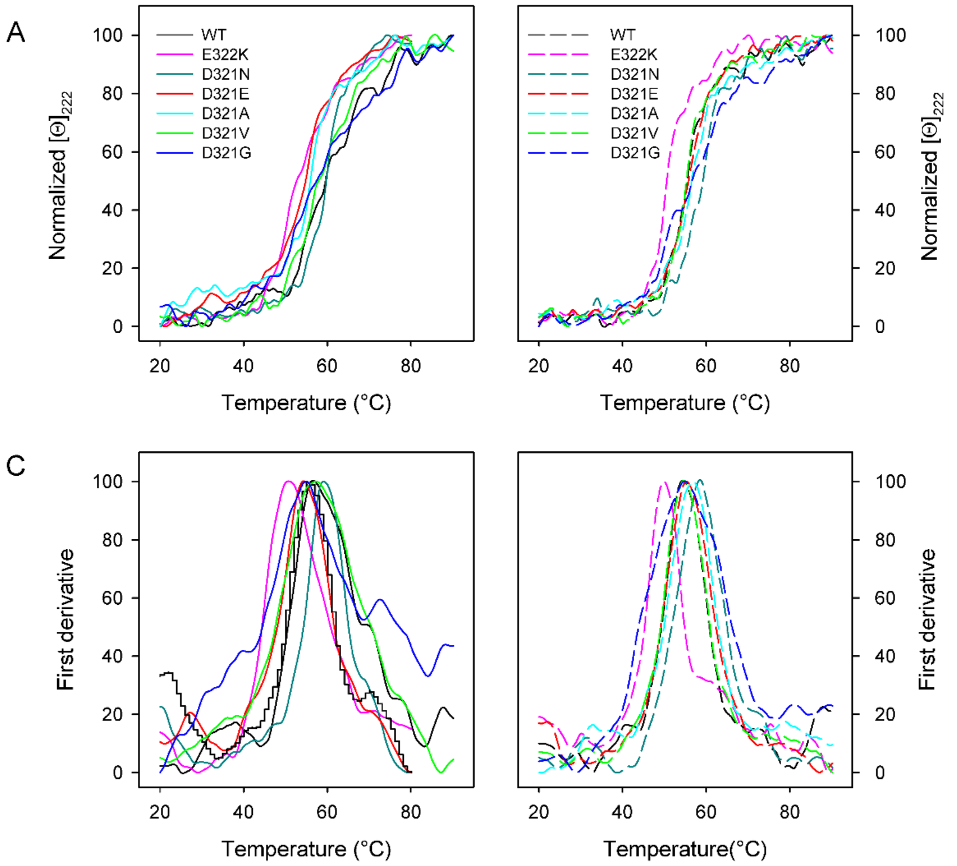

Figure S3. Thermal unfolding of nonphosphorylated-ERK2 (NP-ERK2) and phosphorylated ERK2 (P-ERK2) wild type and variants in the CD-site. ERK2 wild-type and variants (103 µg/ml - 130 µg/ml) were heated from 20°C to 95°C in 20 mM Tris-HCl, pH 8.0 containing 0.2 M NaCl and 0.2 mM DTT. The molar ellipticity at 222 nm ([Θ_{222}]) was monitored continuously every 0.5 °C. (**A**) Thermal unfolding of NP-ERK2 wild type and variants. (**B**) Thermal unfolding of P-ERK2 wild-type and variants. First derivative of NP-ERK2 (**C**) and P-ERK2 (**D**) wild-type and variants data reported in A and B, respectively.

Table S3 Thermodynamic parameters for GdmCl-induced unfolding equilibrium of nonphosphorylated-ERK2 (NP-ERK2) and phosphorylated ERK2 (P-ERK2) wild-type and variants measured by far-UV CD and fluorescence spectroscopy.

	ΔG^{H_2O} (kcal/mol)		*m* (kcal/mol/M)		$[GdmCl]_{0.5}$ (M)	
**ERK2**	**CD ($[\Theta]_{222}$)**	**Fluorescence ($\bar{\lambda}$)**	**CD ($[\Theta]_{222}$)**	**Fluorescence ($\bar{\lambda}$)**	**CD ($[\Theta]_{222}$)**	**Fluorescence ($\bar{\lambda}$)**
**NP-wild-type**	2.51 + 0.22	2.79 + 0.16	0.91 + 0.09	1.34 + 0.08	2.76	2.08
**P- wild-type**	2.30 +0.16	2.88 + 0.20	0.97 + 0.07	1.19 + 0.09	2.37	2.42
**NP-D321A**	2.16 + 0.19	2.91 + 0.20	1.08 + 0.10	1.39 + 0.10	2.00	2.09
**P-D321A**	1.83 + 0.14	2.89 + 0.28	0.90 + 0.07	1.51 + 0.15	2.03	1.92
**NP- D321E**[b]	2.43 + 0.20	ΔG1 = 2.93<br>ΔG2 = 4.76	1.13 + 0.10	m1 = 2.15 + 0.15<br>m2 = 1.41 + 0.38	2.16	[GdmCl]0.5 = 1.36 + 0.02<br>[GdmCl]0.5 = 3.36 + 0.13
**P-D321E**[a]	ΔG1 = 3.39<br>ΔG2 = 6.99	2.09 + 0.13	m1 = 2.50 + 0.39<br>m2 = 1.96 + 0.57	1.13 + 0.10	[Gdm]0.5 = 1.59 + 0.05<br>[Gdm]0.5 = 3.57 + 0.08	2.02
**NP-D321G**[a]	ΔG1 = 2.95<br>ΔG2 = 4.41	2.91 + 0.23	m1 = 2.45 + 0.43<br>m2 = 1.27 + 0.47	1.37 + 0.11	[Gdm]0.5 = 1.20 + 0.04<br>[Gdm]0.5 = 3.48 + 0.21	2.12
**P-D321G**	2.31 + 0.13	2.97 + 0.26	1.01 + 0.07	1.26 + 0.12	2.28	2.35
**NP-D321N**	2.07 + 0.16	2.57 + 0.20	1.00 + 0.08	1.17 + 0.10	2.06	2.20

	ΔG^{H_2O} (kcal/mol)		*m* (kcal/mol/M)		$[GdmCl]_{0.5}$ (M)	
**ERK2**	**CD ($[\Theta]_{222}$)**	**Fluorescence ($\bar{\lambda}$)**	**CD ($[\Theta]_{222}$)**	**Fluorescence ($\bar{\lambda}$)**	**CD ($[\Theta]_{222}$)**	**Fluorescence ($\bar{\lambda}$)**

**P-D321N**[a]	ΔG1 = 2.39<br>ΔG2 = 8.09	3.23 + 0.29	m1 = 2.03 + 0.22<br>m 2 = 2.43 + 0.71	1.38 + 0.13	[Gdm]0.5 = 1.18 + 0.03<br>[Gdm]0.5 = 3.34 + 0.08	2.35
**NP-D321V**	1.26 + 0.10	2.48 + 0.18	0.99 + 0.07	1.08 + 0.08	1.27	2.29
**P-D321V**[a]	ΔG1 = 5.00<br>ΔG2 = 4.92	2.69 + 0.22	m1 = 5.02 + 0.22<br>m2 = 1.56 + 0.25	1.32 + 0.12	[Gdm]0.5 = 1.00 + 0.03<br>[Gdm]0.5 = 3.16 + 0.07	2.29
**NP-E322K**	1.93 + 0.14	2.50 + 0.18	0.76 + 0.06	1.14 + 0.09	2.53	2.19
**P-E322K**[a,b]	ΔG1 = 4.02<br>ΔG2 = 3.97	ΔG1 = 5.08<br>ΔG2 = 5.78	m1 = 4.62 + 0.49<br>m2 = 1.27 + 0.13	m1 = 4.73 + 0.57<br>m 2 = 1.53 + 0.52	[Gdm]0.5 = 0.87 + 0.01<br>[Gdm]0.5 = 3.13 + 0.06	[Gdm]0.5 = 1.07 + 0.02<br>[Gdm]0.5 = 3.77 + 0.27

GdmCl-induced unfolding equilibrium data were measured as described in Materials and Methods by monitoring the ellipticity at 222 nm ($[\Theta_{222}]$) and the fluorescence intensity averaged emission wavelength ($\bar{\lambda}$, Equation (2)). ΔG^{H_2O} and m values were obtained from Equation (3); $[GdmCl]_{0.5}$ was calculated from Equation (5). [a]$[\Theta_{222}]$data were fitted to to Equation (6). [b] $\bar{\lambda}$ data were fitted to Equation (6). Data are reported as the mean ± SE of the fit.

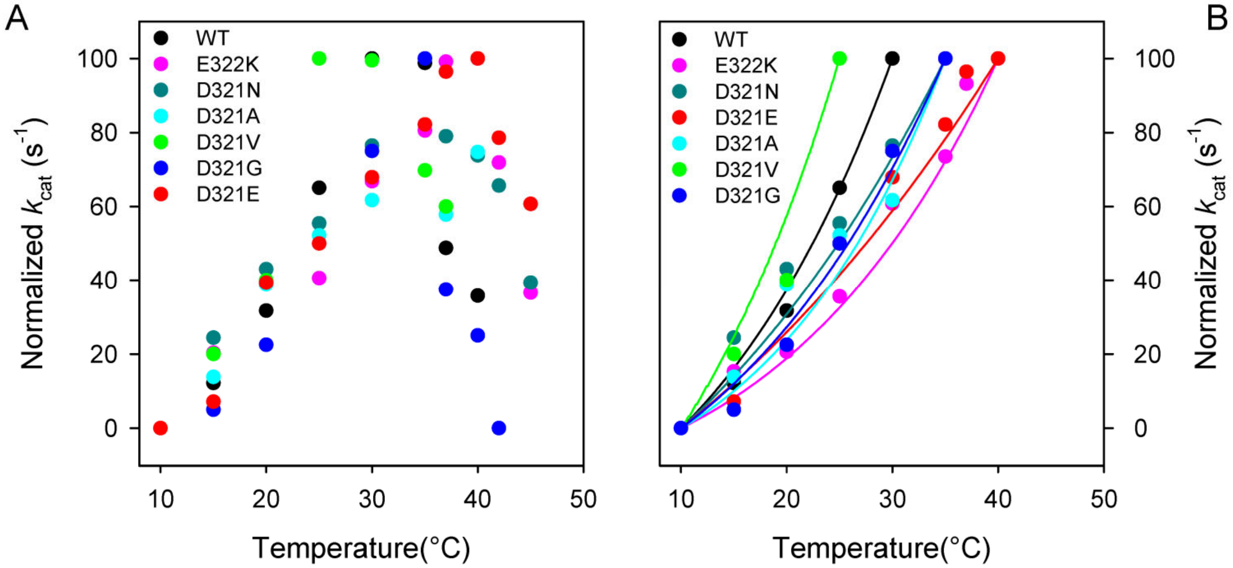

Figure S4. Effect of temperature on the activity of phosphorylated ERK2 (P-ERK2) wild-type and variants. (**A**) Temperature dependence of kinase activity of ERK2 wild-type and variants. (**B**) Non-linear fit of the temperature dependence of ERK2 activity to the Arrhenius equation (Equation (1). Assays were performed under the conditions described in Materials and Methods**.**

Table S4. Selected inhibitors of ERK2. The inhibitors were analyzed by DSF for ERK2 wild-type and variants, as described in Materials and Methods.

Compound	Supplier	Compound Description	Reference
SCH772984	Cayman	ERK inhibitor	[47]
AZD0364	Cayman	ERK inhibitor	[48]
KO-947	Cayman	ERK inhibitor	[49]
LY3214996	Cayman	ERK inhibitor	[50]
FR 180204	Cayman	ERK inhibitor	[51]
GDC-0994	Cayman	ERK inhibitor	[52]
Ulixertinib	Cayman	ERK inhibitor	[53]
Magnolin	Cayman	ERK inhibitor	[54]

Table S5. Effect of the binding with inhibitors on melting temperature of ERK2 wild-type (WT) and variants. The values below 1 suggest that the ligand binds to the protein preferentially to the unfolded state or to non-native states and stabilizes (increases the molar fraction or the population of) the unfolded or non-native states.

	ΔTm (°C)						
**Compound**	**WT**	**D321A**	**D321E**	**D321G**	**D321N**	**D321V**	**E322K**
**AZD0346**	12.80 ± 0.05	13.30 ± 0.51	13.70 ± 1.03	13.90 ± 0.89	14.50 ± 0.21	13.00 ± 0.03	10.20 ± 0.01
**KO-947**	11.80 ± 0.22	12.30 ± 0.37	12.80 ± 1.01	13.10 ± 0.86	13.60 ± 0.23	11.80 ± 0.02	9.20 ± 0.08
**SCH772984**	11.60 ± 0.27	11.00 ± 0.09	11.70 ± 1.15	12.20 ± 0.89	12.70 ± 0.24	11.60 ± 0.13	8.70 ± 0.15
**GDC-0994**	10.30 ± 0.30	10.70 ± 0.20	11.20 ± 0.74	11.80 ± 0.58	12.20 ± 0.15	10.30 ± 0.28	6.80 ± 0.49
**LY3214996**	9.40 ± 0.21	9.80 ± 0.02	10.30 ± 0.88	10.80 ± 0.67	11.20 ± 0.14	10.00 ± 0.02	6.60 ± 0.01
**Ulixertinib**	7.90 ± 0.39	8.30 ± 0.31	8.80 ± 0.95	9.10 ± 0.79	9.50 ± 0.24	8.30 ± 0.09	4.60 ± 0.14
**FR 180204**	2.80 ± 1.27	3.20 ± 0.20	3.70 ± 0.86	4.60 ± 0.94	5.40 ± 0.58	5.00 ± 0.44	-1.30 ± 0.08
**Magnolin**	0.20 ± 0.11	0.60 ± 0.24	1.40 ± 0.43	1.90 ± 1.13	2.60 ± 0.35	0.30 ± 0.10	-5.30 ± 0.35
